# Supplementary material for: Flexible computation of object motion and depth based on viewing geometry inferred from optic flow
Source: Nat Commun. 2025 Dec 30;17:1092. doi: 10.1038/s41467-025-67857-4 (PMC12852675; doi:10.1038/s41467-025-67857-4)
Supplement: Supplementary file 1 — Supplementary Information [file 41467_2025_67857_MOESM1_ESM.pdf]

Flexible computation of object motion and depth based on viewing geometry inferred from optic flow

Supplementary Information

Supplementary Table

Participants	a_{ret}, Pursuit		a_{ret}, Fixation		a_{eye}, Pursuit		a_{eye}, Fixation	
	p	Δm	p	Δm	p	Δm	p	Δm
h201	1.27×10^{-83}	-0.460	1.39×10^{-83}	-0.577	1.26×10^{-83}	0.459	1.27×10^{-83}	0.367
h500	1.27×10^{-83}	-0.602	1.83×10^{-81}	-0.373	1.26×10^{-83}	0.331	1.27×10^{-83}	0.274
h501	1.14×10^{-4}	-0.000	4.63×10^{-82}	0.518	1.26×10^{-83}	0.378	1.27×10^{-83}	0.254
h507	1.35×10^{-77}	-0.376	1.27×10^{-83}	-0.473	1.27×10^{-83}	0.509	1.27×10^{-83}	0.481
h510	1.05×10^{-74}	-0.000	1.27×10^{-83}	-0.269	1.27×10^{-83}	0.353	1.27×10^{-83}	0.416
h512	2.21×10^{-83}	-0.195	5.47×10^{-5}	0.000	1.27×10^{-83}	0.224	1.27×10^{-83}	0.437
h518	1.27×10^{-83}	-0.537	1.27×10^{-83}	-0.705	1.27×10^{-83}	0.335	1.27×10^{-83}	0.377
h520	0.123	0.000	1.38×10^{-83}	0.496	1.27×10^{-83}	0.196	1.27×10^{-83}	0.302
h521	1.49×10^{-83}	-0.313	1.29×10^{-20}	0.207	1.27×10^{-83}	0.206	1.27×10^{-83}	0.243

Table S1: Statistics of model parameters between R and R+T conditions for each participant in Experiment 1 (p-values and difference of medians; two-sided Wilcoxon signed-rank test; N=500 bootstrap resamples).

Supplementary Figures

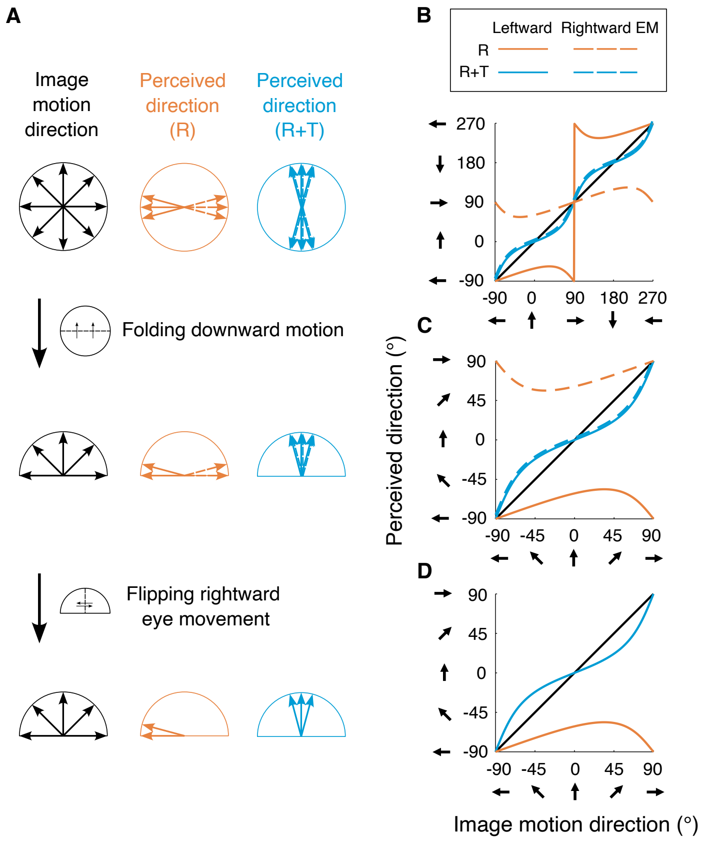

Figure S1: Schematics of data folding for Experiment 1. **A**, Our model predicts that in both viewing geometries, perceived motion directions are symmetric about the horizontal axis (top panel). Accordingly, data for downward motion were folded upward to merge with the upward motion directions (middle panel). Furthermore, because perceived directions under opposite eye movements are vertically symmetric, data for rightward eye movements were flipped to merge with those for leftward eye movements (bottom panel). Note that folding was performed only for visualization, and not for model fitting. **B**-**D**, Relationship between perceived direction and image motion direction at each folding step illustrated in **A**.

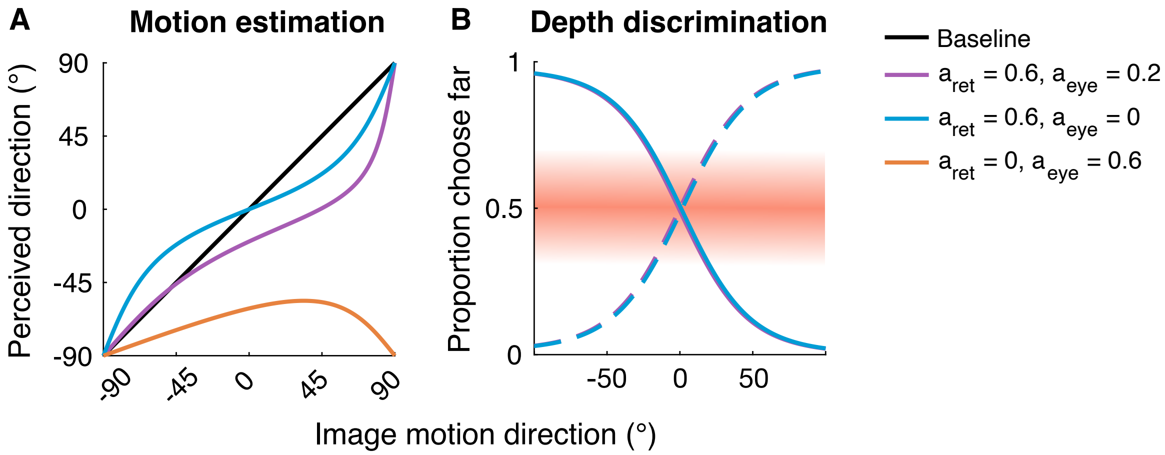

Figure S2: Model predictions for Experiments 1 (**A**) and 2 (**B**) with different values of a_{ret} and a_{eye}. **A**, Canonical predictions for motion perception in the R (orange) and R+T (blue) geometries have $a_{\text{ret}} = 0$ and $a_{\text{eye}} = 0$, respectively. When a value of $a_{\text{eye}} > 0$ is applied to the R+T prediction (purple), the curve shifts downward, consistent with data observed from several participants in the Pursuit condition. **B**, Adding a value of $a_{\text{eye}} > 0$ to the R+T geometry prediction for the depth perception task has no effect on the expected psychometric functions. Overlapping blue and purple curves in panel B have been shifted very slightly for visibility.

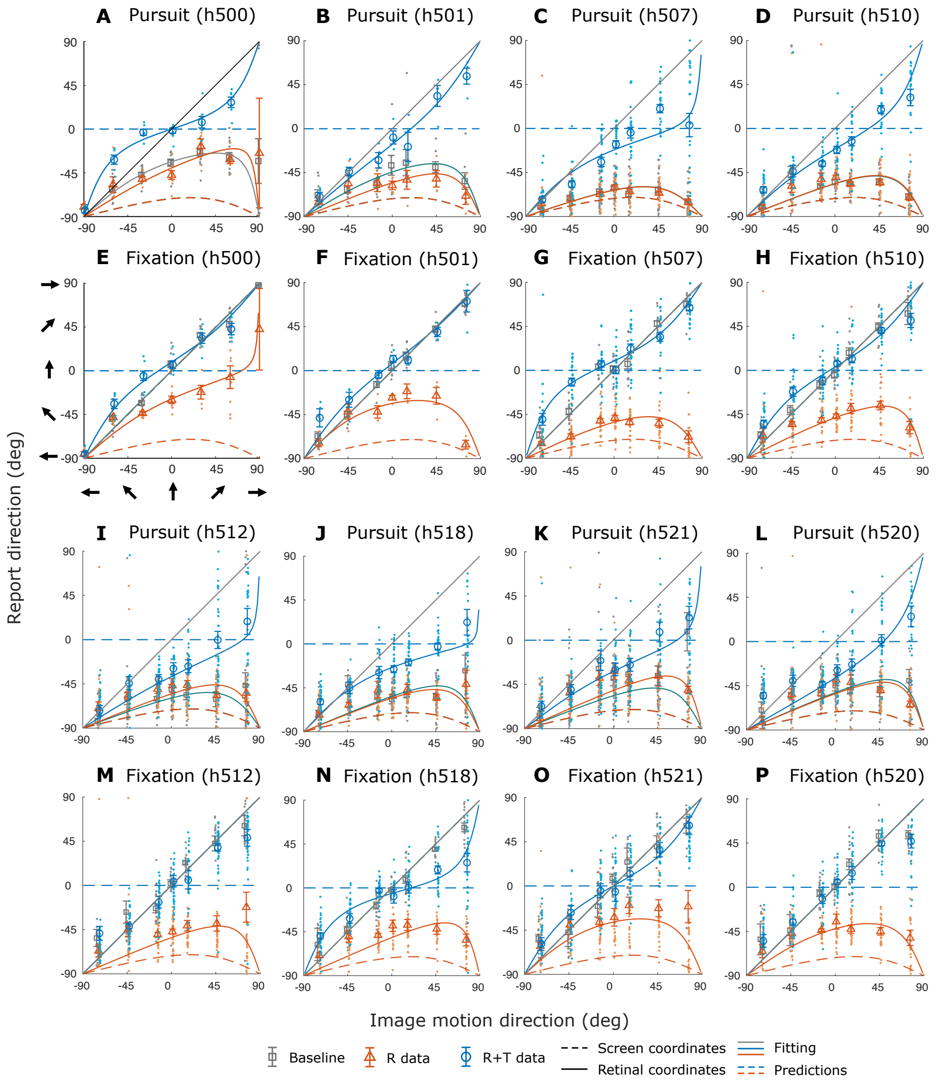

Figure S3: Results of the motion estimation task from all participants, except for the example participant (h201) already shown in Figure 6. **A-D & I-L**, Data from the Pursuit condition for eight additional participants. **E-H & M-P**, Data from the Fixation condition for the same participants. N=24 independent trials for each condition. Format as in Figure 6D and E. All participants shown are included in Figure 6G-H and all statistical analyses.

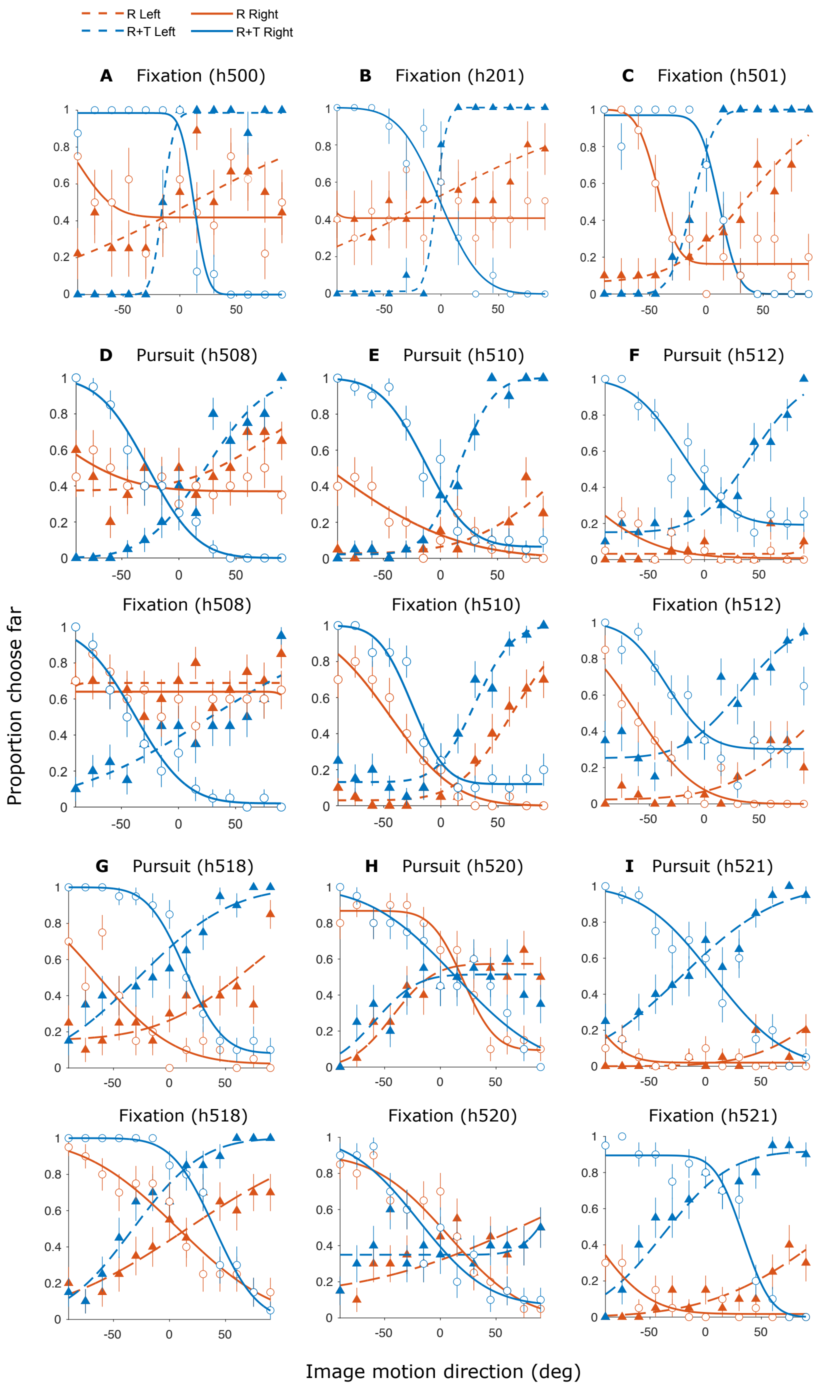

Figure S4: Results from the depth discrimination task from all participants, except for the example participant (h507) already shown in Figure 7. **A-C**, Results from three non-naïve participants in the Fixation condition. **D-I**, Results from naïve participants in the Pursuit (top) and Fixation (bottom) conditions. N=20 independent trials for each condition. Format as in Figure 7D and E. All participants shown are included in Figure 7G-H and all statistical analyses.

Supplementary Methods

Derivation of viewing geometry

Consider a general scenario in which the observer translates their body (or head) laterally while tracking a moving fixation target by pursuit eye movements with an angular velocity relative to the scene, ω_{eye} (Figure 3). Meanwhile, another object moves independently in the frontoparallel plane at a certain distance, d, from the fixation target.

The retinal motion, ω_{ret}, of the object has three components: (1) image motion produced by object motion in the world, ω_{obj}, (2) motion parallax produced by observer's translation, ω_{ret}^{T}, and (3) image motion produced by the eye rotation that tracks the moving fixation target, ω_{ret}^{P} (Figure 3):

$$\omega_{\text{ret}} = \omega_{\text{obj}} + \omega_{\text{ret}}^{T} + \omega_{\text{ret}}^{P}. \tag{24}$$

Rewriting the approximate form of the motion-pursuit law[1], $d/f = \omega_{\text{ret}}^{T}/\omega_{\text{eye}}^{T}$, the motion parallax component is computed as:

$$\omega_{\text{ret}}^{T} = \frac{d}{f}\omega_{\text{eye}}^{T}, \tag{25}$$

where f is the viewing distance and ω_{eye}^{T} is the angular velocity of the eye rotation (relative to the scene) that compensates for the eye's translation relative to the scene (as opposed to the eye rotation needed to track a moving fixation target).

To obtain ω_{eye}^{T}, consider the intersection at a distance, p, between the line of sight at the initial time point t_0 and that at a later time point $t_0 + dt$. The position of p describes the relationship between the movement of the fixation target and that of the observer, and $p/f = \tan(\omega_{\text{eye}}^{T})/\tan(\omega_{\text{eye}})$, where ω_{eye} is the angular velocity of

the total eye rotation. For small angles, $\tan(\omega) \approx \omega$, thus ω_{eye}^{T} can be computed as:

$$\omega_{\text{eye}}^{T} = \frac{p}{f}\omega_{\text{eye}}. \tag{26}$$

Note that while the distance between the rotation pivot and the eye, p, can change as the eye translates, the ratio between p and f remains constant for lateral translations (Figure 3).

The third component of retinal motion, ω_{ret}^{P}, is the opposite of the eye velocity caused by a moving fixation target, ω_{eye}^{P}:

$$\omega_{\text{ret}}^{P} = -\omega_{\text{eye}}^{P}. \tag{27}$$

Because $\omega_{\text{eye}}^{P} + \omega_{\text{eye}}^{T} = \omega_{\text{eye}}$, ω_{ret}^{P} can be computed as:

$$\omega_{\text{ret}}^{P} = -\left(1 - \frac{p}{f}\right)\omega_{\text{eye}}. \tag{28}$$

From Equations (24) to (28), we can obtain the angular velocity of the object as:

$$\omega_{\text{obj}} = \omega_{\text{ret}} + \left(1 - \left(1 + \frac{d}{f}\right)\frac{p}{f}\right)\omega_{\text{eye}}. \tag{29}$$

Normalizing the object's depth, d, and the rotation pivot, p, by viewing distance, f, we have:

$$\omega_{\text{obj}} = \omega_{\text{ret}} + \left(1 - \left(1 + d'\right)p'\right)\omega_{\text{eye}}, \tag{30}$$

where $d' \triangleq d/f$ and $p' \triangleq p/f$. In the absence of another depth cue, ω_{obj} and d' are underdetermined, even if p' is specified.

Notably, although we use the approximate formula for the motion-pursuit law here (Equation 4;[1]), Equation (2) still applies if we replace d' with a more accurate form,

$d' \triangleq d/(d+f)$. It is also worth noting that we only consider scenarios in which the observer, pursuit target, and object translate in the fronto-parallel plane, as depicted in Figure 3. When the pursuit target moves in depth, the rotation pivot p' is not constant and Equation (2) no longer applies.

Details of stimulus generation

To ensure that the motion of the object on the screen was the same across the two viewing geometries, we derived the relationship between the simulated 3D geometry in OpenGL and the screen projections based on standard projective geometry. The 3D coordinates of the object and camera were then determined by back-tracing from the desired image positions and motion.

Projective geometry. 3D coordinates in a virtual OpenGL environment can be converted to a normalized screen coordinate system in two steps[2,3]. First, multiply the 3D coordinates, $\mathbf{X} = \begin{pmatrix} X \\ Y \\ Z \\ 1 \end{pmatrix}$, with a perspective projection matrix, $\mathbf{P}$:

$$\mathbf{Y} = \mathbf{P}\mathbf{X}. \tag{31}$$

Here, the X-axis is pointing from left to right, Y-axis is pointing upward, Z-axis is pointing forward, and the projection matrix is given by:

$$\mathbf{P} = \begin{pmatrix} \frac{2Z_{\text{near}}}{s_W} & & & \\ & \frac{2Z_{\text{near}}}{s_H} & & \\ & & -\frac{(Z_{\text{far}}+Z_{\text{near}})}{Z_{\text{far}}-Z_{\text{near}}} & -\frac{2Z_{\text{far}}Z_{\text{near}}}{Z_{\text{far}}-Z_{\text{near}}} \\ & & -1 & \end{pmatrix}, \tag{32}$$

where Z_{near} and Z_{far} are the z-coordinates of the near and far clipping planes, respectively. In our experiments, $Z_{\text{near}} = 5$ cm and $Z_{\text{far}} = 150$ cm. s_W and s_H are the width and height of the screen, respectively, and $s_W = 105.2$ cm, $s_H = 59.2$ cm. The resultant homogeneous coordinates are $\mathbf{Y} = \begin{pmatrix} x \\ y \\ z \\ w \end{pmatrix}$. Homogeneous coordinates were used so that 3D projection, translation, and rotation can be conveniently written as matrix multiplications[3]. The fourth dimension, w, allowed us to represent points at infinity easily as $w = 0$. When $w = 1$, homogeneous coordinates would be the same as 3D Cartesian coordinates. This conversion is conventional in computer graphics including the OpenGL library we used[2,3].

Next, we can obtain the normalized screen coordinates, $\mathbf{N}$:

$$\mathbf{N} = \frac{1}{w}\mathbf{Y} = \frac{1}{w}\mathbf{PX}, \tag{33}$$

and $w = -Z$.

Object and object motion. Now consider an object whose location on the screen is defined as (x_0, y_0). Normalizing its screen coordinates by screen size, we have:

$$\mathbf{N} = \begin{pmatrix} 2x_0/s_W \\ 2y_0/s_H \\ z_n \\ 1 \end{pmatrix} \tag{34}$$

Object motion in the world is essentially a linear transformation, $\mathbf{M}$, on the 3D coordinates, $\mathbf{X}$. Consider only translation along three axes, T_X^{obj}, T_Y^{obj}, and T_Z^{obj}:

$$\mathbf{M} = \begin{pmatrix} 1 & & & T_X^{\text{obj}} \\ & 1 & & T_Y^{\text{obj}} \\ & & 1 & T_Z^{\text{obj}} \\ & & & 1 \end{pmatrix} \tag{35}$$

Self-motion. Similarly, self-motion is another linear transformation, $\mathbf{V}$, on 3D coordinates. In this study, we only consider the rotation of the eye (i.e., rotation θ about the y-axis) and translation of the eye/head on a horizontal plane (i.e., translation along x- and z-axes, T_X^{cam} and T_Z^{cam}):

$$\mathbf{V} = \begin{pmatrix} \cos\theta & & \sin\theta & \\ & 1 & & \\ -\sin\theta & & \cos\theta & \\ & & & 1 \end{pmatrix} \begin{pmatrix} 1 & & & -T_X^{\text{cam}} \\ & 1 & & \\ & & 1 & -T_Z^{\text{cam}} \\ & & & 1 \end{pmatrix} \tag{36}$$

$$= \begin{pmatrix} \cos\theta & & \sin\theta & -T_X^{\text{cam}}\cos\theta - T_Z^{\text{cam}}\sin\theta \\ & 1 & & \\ -\sin\theta & & \cos\theta & T_X^{\text{cam}}\sin\theta - T_Z^{\text{cam}}\cos\theta \\ & & & 1 \end{pmatrix} \tag{37}$$

Retinal motion. Retinal motion is defined as a translation on the screen, with a certain amplitude, l, and direction, α. Therefore, the desired linear transformation, $\mathbf{A}$, in screen coordinates is:

$$\mathbf{A} = \begin{pmatrix} 1 & & & 2l\cos\alpha/s_W \\ & 1 & & 2l\sin\alpha/s_H \\ & & 1 & \\ & & & 1 \end{pmatrix} \tag{38}$$

To present the desired retinal motion, $\mathbf{A}$, on the screen, we need to find the correct 3D coordinates, $\mathbf{X}$, object motion, $\mathbf{M}$, and self-motion, $\mathbf{V}$, such that:

$$\begin{cases} \mathbf{N} & = \frac{1}{w}\mathbf{P}\mathbf{X} \\ \mathbf{A}\mathbf{N} & = \frac{1}{w}\mathbf{P}\mathbf{V}\mathbf{M}\mathbf{X}. \end{cases} \tag{39}$$

Here, the first equation specifies a mapping from world coordinates to normalized screen coordinates at the beginning of the trial t_0, the second equation specifies such a mapping at a later time point $t_0 + \Delta t$, $\mathbf{P}$ is constant across time, $\mathbf{V}$ and $\mathbf{M}$ are the self-motion and object motion during time interval Δt, respectively. Therefore, by solving the equations we can make sure that both viewing geometries produce the desired retinal motion.

Solution for the R viewing geometry. In the R geometry, the observer's eye rotates around the y-axis and does not translate; therefore $T_X^{\text{cam}} = T_Z^{\text{cam}} = 0$. The initial z-coordinate of the object, Z, is the viewing distance, $Z = -f$. Solving for Equation (39), we have:

$$
\begin{cases}
w = f, \\
X = \frac{f}{Z_{\text{near}}} x_0, \\
Y = \frac{f}{z_{\text{near}}} y_0, \\
z_n = \left(-2Z_{\text{far}} Z_{\text{near}}/f + Z_{\text{far}} + Z_{\text{near}}\right) / \left(Z_{\text{far}} - Z_{\text{near}}\right), \\
T_X^{\text{obj}} = \frac{f}{Z_{\text{near}}} \left(x_0 \left(\cos\theta - 1\right) + l\cos\alpha\right) + f\sin\theta, \\
T_Y^{\text{obj}} = \frac{fl\sin\alpha}{Z_{\text{near}}}, \\
T_Z^{\text{obj}} = \frac{f}{Z_{\text{near}}} (x_0 + \frac{l\cos\alpha}{\cos\theta}) \sin\theta + f(1 - \cos\theta).
\end{cases}
\tag{40}
$$

Solution of the R+T viewing geometry. In the R+T geometry, the object is located at a known depth, Z, and the observer's eye translates along the x-axis while counter-rotating about the y-axis to maintain fixation, therefore $\frac{T_X^{\text{cam}}}{f - T_Z^{\text{cam}}} = \tan\theta$. The object only moves along the y-axis, thus $T_X^{\text{obj}} = T_Z^{\text{obj}} = 0$. Solving for Equation (39):

$$
\begin{cases}
w = -Z, \\
Z = -fZ_{\text{near}}\sin\theta / \left(x_0 \left(\cos\theta - 1\right) - l\cos\alpha - Z_{\text{near}}\sin\theta\right), \\
X = -\frac{Z}{Z_{\text{near}}} x_0, \\
Y = -\frac{Z}{Z_{\text{near}}} y_0, \\
z_n = \left(2Z_{\text{far}} Z_{\text{near}}/Z + Z_{\text{far}} + Z_{\text{near}}\right) / \left(Z_{\text{far}} - Z_{\text{near}}\right), \\
T_Z^{\text{cam}} = f\sin^2\theta + Z\cos\theta(\cos\theta + \frac{x_0}{Z_{\text{near}}}\sin\theta - 1), \\
T_X^{\text{cam}} = (f - T_Z^{\text{cam}})\tan\theta, \\
T_Y^{\text{obj}} = -\frac{Z}{Z_{\text{near}}} l\sin\alpha.
\end{cases}
\tag{41}
$$

Supplementary References

1. Nawrot, M. & Stroyan, K. The motion/pursuit law for visual depth perception from motion parallax. *Vision Research* **49,** 1969–1978 (2009).

2. Woo, M., Neider, J., Davis, T. & Shreiner, D. *OpenGL programming guide: the official guide to learning OpenGL, version 1.2* (Addison-Wesley Longman Publishing Co., Inc., 1999).

3. Bloomenthal, J. & Rokne, J. Homogeneous coordinates. *The Visual Computer* **11,** 15–26 (1994).
