# Supplementary material for: Flexible computation of object motion and depth based on viewing geometry inferred from optic flow
Source: Nat Commun. 2025 Dec 30;17:1092. doi: 10.1038/s41467-025-67857-4 (PMC12852675; doi:10.1038/s41467-025-67857-4)
Supplement: Supplementary file 2 — Reporting Summary [file 41467_2025_67857_MOESM2_ESM.pdf]

nature portfolio

Corresponding author(s): Zhe-Xin Xu

Last updated by author(s): Nov 21, 2025

Reporting Summary

Nature Portfolio wishes to improve the reproducibility of the work that we publish. This form provides structure for consistency and transparency in reporting. For further information on Nature Portfolio policies, see our Editorial Policies and the Editorial Policy Checklist.

Statistics

For all statistical analyses, confirm that the following items are present in the figure legend, table legend, main text, or Methods section.

n/a	Confirmed	
☐	☒	The exact sample size (*n*) for each experimental group/condition, given as a discrete number and unit of measurement
☐	☒	A statement on whether measurements were taken from distinct samples or whether the same sample was measured repeatedly
☐	☒	The statistical test(s) used AND whether they are one- or two-sided<br>*Only common tests should be described solely by name; describe more complex techniques in the Methods section.*
☐	☒	A description of all covariates tested
☒	☐	A description of any assumptions or corrections, such as tests of normality and adjustment for multiple comparisons
☐	☒	A full description of the statistical parameters including central tendency (e.g. means) or other basic estimates (e.g. regression coefficient) AND variation (e.g. standard deviation) or associated estimates of uncertainty (e.g. confidence intervals)
☐	☒	For null hypothesis testing, the test statistic (e.g. *F*, *t*, *r*) with confidence intervals, effect sizes, degrees of freedom and *P* value noted<br>*Give P values as exact values whenever suitable.*
☒	☐	For Bayesian analysis, information on the choice of priors and Markov chain Monte Carlo settings
☒	☐	For hierarchical and complex designs, identification of the appropriate level for tests and full reporting of outcomes
☐	☒	Estimates of effect sizes (e.g. Cohen's *d*, Pearson's *r*), indicating how they were calculated

Our web collection on statistics for biologists contains articles on many of the points above.

Software and code

Policy information about availability of computer code

Data collection	Data were collected using commercial software TEMPO (Reflective Computing, Olympia WA)
Data analysis	Data were analyzed using custom MATLAB code available in OSF: https://doi.org/10.17605/OSF.IO/ZY8W6

For manuscripts utilizing custom algorithms or software that are central to the research but not yet described in published literature, software must be made available to editors and reviewers. We strongly encourage code deposition in a community repository (e.g. GitHub). See the Nature Portfolio guidelines for submitting code & software for further information.

Data

Policy information about availability of data

All manuscripts must include a data availability statement. This statement should provide the following information, where applicable:

- Accession codes, unique identifiers, or web links for publicly available datasets
- A description of any restrictions on data availability
- For clinical datasets or third party data, please ensure that the statement adheres to our policy

The experimental data are available at: https://doi.org/10.17605/OSF.IO/ZY8W6

Research involving human participants, their data, or biological material

Policy information about studies with human participants or human data. See also policy information about sex, gender (identity/presentation), and sexual orientation and race, ethnicity and racism.

Reporting on sex and gender	Gender of each participant was determined based on self-report. Overall number of each gender was reported in the Methods section. Sex and gender were not relevant for our study and were not analyzed separately.
Reporting on race, ethnicity, or other socially relevant groupings	No race, ethnicity, or other socially relevant groupings were used in our study.
Population characteristics	See below
Recruitment	Human subjects were recruited via a dedicated email list at the University of Rochester. Self selection bias may be present because volunteers could differ from non participants in interest or motivation, therefore exhibit differences in lapse rate and perceptual threshold measured in the study. However, it is unlikely that these self-selection biases will impact our results, which focused on comparisons between experimental conditions.
Ethics oversight	This study was approved by the Office of Human Subject Protection at the University of Rochester

Note that full information on the approval of the study protocol must also be provided in the manuscript.

Field-specific reporting

Please select the one below that is the best fit for your research. If you are not sure, read the appropriate sections before making your selection.

☐ Life sciences ☒ Behavioural & social sciences ☐ Ecological, evolutionary & environmental sciences

For a reference copy of the document with all sections, see nature.com/documents/nr-reporting-summary-flat.pdf

Behavioural & social sciences study design

All studies must disclose on these points even when the disclosure is negative.

Study description	All data are quantitative measures, including button pressing behavior and analog report by turning a dial
Research sample	Participants were students, staff, or faculty members of the University of Rochester, including 4 males and 6 females, 18-58 years old. The sample size may not be large enough to be considered representative of the general population. Participants were chosen based on voluntary response to a recruitment email.
Sampling strategy	Voluntary response sampling was used in this study because participants were recruited via university email list. No sample size calculation was performed, and the sample size was chosen based on the number of voluntary responses we received. The sample size is sufficient because each participant completed a large number of trials and exhibited similar patterns.
Data collection	Participants viewed visual stimuli on a computer screen and reported either the motion direction of an object by turning a dial or the depth of an object by pressing a button. Eye movements were recorded using an Eyelink eye tracker. No one else was present during the experiments. The researchers were blinded to the experimental conditions because the condition in each trial was randomly chosen by the computer.
Timing	Data were collected between 6/17/2021 and 3/27/2024
Data exclusions	No data were excluded from analyses
Non-participation	One participant dropped out because of relocation
Randomization	Participants were not allocated into groups

Reporting for specific materials, systems and methods

We require information from authors about some types of materials, experimental systems and methods used in many studies. Here, indicate whether each material, system or method listed is relevant to your study. If you are not sure if a list item applies to your research, read the appropriate section before selecting a response.

Materials & experimental systems

n/a	Involved in the study
☒	☐ Antibodies
☒	☐ Eukaryotic cell lines
☒	☐ Palaeontology and archaeology
☒	☐ Animals and other organisms
☒	☐ Clinical data
☒	☐ Dual use research of concern
☒	☐ Plants

Methods

n/a	Involved in the study
☒	☐ ChIP-seq
☒	☐ Flow cytometry
☒	☐ MRI-based neuroimaging

Plants

Seed stocks

Report on the source of all seed stocks or other plant material used. If applicable, state the seed stock centre and catalogue number. If plant specimens were collected from the field, describe the collection location, date and sampling procedures.

Novel plant genotypes

Describe the methods by which all novel plant genotypes were produced. This includes those generated by transgenic approaches, gene editing, chemical/radiation-based mutagenesis and hybridization. For transgenic lines, describe the transformation method, the number of independent lines analyzed and the generation upon which experiments were performed. For gene-edited lines, describe the editor used, the endogenous sequence targeted for editing, the targeting guide RNA sequence (if applicable) and how the editor was applied.

Authentication

Describe any authentication procedures for each seed stock used or novel genotype generated. Describe any experiments used to assess the effect of a mutation and, where applicable, how potential secondary effects (e.g. second site T-DNA insertions, mosiacism, off-target gene editing) were examined.
